# Fatty alcohols, C_12–18_: MAK Value Documentation – Translation of the German version from 2024

**DOI:** 10.34865/mb6776225kske11_1or

**Published:** 2026-03-30

**Authors:** Andrea Hartwig, MAK Commission

**Affiliations:** 1 Institute of Applied Biosciences, Department of Food Chemistry and Toxicology, Karlsruhe Institute of Technology (KIT) Adenauerring 20a, Building 50.41 76131 Karlsruhe Germany; 2 Permanent Senate Commission for the Investigation of Health Hazards of Chemical Compounds in the Work Area, Deutsche Forschungsgemeinschaft, Kennedyallee 40, 53175 Bonn, Germany. Further information: Permanent Senate Commission for the Investigation of Health Hazards of Chemical Compounds in the Work Area | DFG

**Keywords:** fatty alcohols, C_12–18_, irritation, respiratory tract, emulsifier, sensitization, Luft, air

## Abstract

The German Senate Commission for the Investigation of Health Hazards of Chemical Compounds in the Work Area (MAK Commission) summarized and evaluated the data for C_12–18_ fatty alcohols [67762-25-8] to derive an occupational exposure limit value (maximum concentration at the workplace, MAK value) considering all toxicological end points. Relevant studies were identified from a literature search. There are no data for humans or repeated dose inhalation studies in animals to derive a MAK value for the C_12–18_ fatty alcohols. Oral studies investigating alcohols with similar chain length resulted in NOAELs which would correspond to a concentration of 92 mg/m^3^ or higher at the workplace. However, as the C_12–18_ fatty alcohols may act as non-ionic surfactants, effects on the pulmonary surfactant are likely to occur. As a result, a MAK value cannot be established. The C_12–18_ fatty alcohols are not mutagenic or clastogenic in vitro. No in vivo genotoxicity studies and no carcinogenicity studies have been carried out with the C_12–18_ fatty alcohols. There are no studies investigating the developmental toxicity of the C_12–18_ fatty alcohols. A sensitizing potential is not expected based on the available data. The substance does not penetrate the skin in toxicologically relevant amounts.

**Table:** 

**MAK value**	**not established, see Section II b of the List of MAK and BAT Values**
**Peak limitation**	**–**
	
**Absorption through the skin**	**–**
**Sensitization**	**–**
**Carcinogenicity**	**–**
**Prenatal toxicity**	**–**
**Germ cell mutagenicity**	**–**
	
**BAT value**	**–**
	
Synonyms	(C_12_–C_18_) alkyl alcohols
Chemical name (IUPAC)	–
CAS number	67762-25-8
Molecular formula	CH_3_(CH_2_)_n_CH_2_OH, n = 10, 12, 14, 16, 100% linear
Molar mass	186–242 g/mol
Melting point	no data, oily liquid or soft mass (IFA 2020)
Boiling point at 1013 hPa	260–350 °C (OECD 2006)
Density at 40 °C	0.81–0.82 g/cm^3^ (OECD 2006)
Vapour pressure at 25 °C	type A^a)^: 0.0016 hPa (calculated) (OECD 2006) type B^b)^: 0.00026 hPa (calculated) (OECD 2006)
log K_OW_ at 25 °C	5.4–7.2 (calculated) (OECD 2006) 6.36 for 1-tetradecanol (IFA 2020)
Solubility	0.35–1.7 mg/l water (calculated) at 25 °C (OECD 2006) 1.3 mg/l water for 1-tetradecanol at 23 °C (IFA 2020)
	
Hydrolytic stability	does not undergo hydrolysis in water (OECD 2006)
Stability	no data
Production	aliphatic alcohols in general: oleochemical from plant or animal-based oils or fats such as coconut oil, palm kernel oil, tallow fat or other triglycerides; petrochemical or from other synthetic processes (OECD 2006)
Purity	95% C_12, 14, 16, 18_ linear alcohols (OECD 2006)
Impurities	5% C_8–10_ and C_20_ linear alcohols such as decanol; do not contain impurities with a chemical structure different from the definition of the category (OECD 2006)
Uses	direct use in paints, lubricants, paper, plastic, textiles, leather, plaster, formwork oils, household products and personal care/cosmetic products, intermediates, cleaners and laundry detergents, additives (OECD 2006)
Concentrations used	1-dodecanol, 1-tetradecanol, 1-hexadecanol, 1-octadecanol concentrations in metal-working fluid concentrates: maximum 5% (Hartwig and MAK Commission 2023, available in German only) Generally 10%, in non-water-miscible metal-working fluids up to 100% (Hartwig and MAK Commission 2023)

^a)^**type A**: > 50% C_12/14_, > 10% C_16/18_, range C_8_ to C_20_ with an even number of C atoms

^b)^**type B**: > 10% C_12/14_, > 60% C_16/18_, range C_12_ to C_20_ with an even number of C atoms (OECD 2006)

Note: The substance can occur simultaneously as vapour and aerosol.

Documentation is available for a number of linear alcohols (see Table 1).

**Tab. 1:** Fatty alcohols included in the List of MAK and BAT Values (DFG 2022)

**Molecular formula**	**Name**	**Common name, MAK value**	**CAS No.**	**References**
C_6_H_13_OH	1-hexanol	caproic alcohol, Section II b	111-27-3	Henschler 1992, available in German only
C_8_H_17_OH	1-octanol	capryl alcohol, 10 ml/m^3^	111-87-5	Greim 2003
C_10_H_21_OH	1-decanol	caprinic alcohol, 10 ml/m^3^	112-30-1	Hartwig and MAK Commission 2022
C_12_H_25_OH	1-dodecanol	lauryl alcohol, Section II b	112-53-8	Greim 2000, available in German only
C_14_H_29_OH	1-tetradecanol	myristyl alcohol, Section II b	112-72-1	Greim 2001 c, available in German only
C_16_H_33_OH	1-hexadecanol	cetyl alcohol, Section II b	36653-82-4	Greim 2001 a, available in German only
C_18_H_37_OH	1-octadecanol	stearyl alcohol, Section II b	112-92-5	Greim 2001 b, available in German only

## 1 Toxic Effects and Mode of Action

The C_12–18_ fatty alcohols are saturated compounds. They are absorbed via all routes of exposure, have low systemic toxicity and occur naturally in human metabolism. Inhalation studies with C_12–18_ fatty alcohols are not available. The fatty alcohols may have a surfactant-like (emulsifying) effect on cell membranes of the respiratory tract if they occur in high concentrations in the air. Fatty alcohols with chains of 12 or more carbon atoms cause slight irritation of the skin of rabbits.

The liver weights and, in some cases, the liver enzymes in the blood were increased after oral administration of longer chain alcohols, such as C_14–16_ or C_16_, for 13 weeks in doses of at least 1000 mg/kg body weight and day; no histopathological findings were detected in the liver.

Constituents of the mixture of C_12–18_ fatty alcohols are weakly sensitizing. The number of contact allergies attributed to C_12–18_ fatty alcohols is considered low compared to their very widespread use. C_12–18_ fatty alcohols are not mutagenic in bacteria. There are no data available for the carcinogenicity of C_12–18_ fatty alcohols but there is also no structural alert. Studies that specifically investigated prenatal toxicity are not available.

## 2 Mechanism of Action

The C_12–18_ fatty alcohols did not cause substance-specific effects in the evaluated studies; however, there were no studies available that investigated the effects on the respiratory tract after long periods of inhalation exposure. As the fatty alcohols have low vapour pressure, the most likely form of exposure at certain workplaces is vapour/aerosol exposure. In this exposure scenario, the C_12–18_ fatty alcohols may induce surfactant-like effects on cell membranes of the respiratory tract via the hydrophilic alcohol end group and the hydrophobic hydrocarbon group of the molecule.

## 3 Toxicokinetics and Metabolism

### 3.1 Absorption, distribution, elimination

Long-chain alcohols are absorbed by varying degrees via all routes of exposure. Fatty alcohols occur naturally in living organisms (OECD 2006; Veenstra et al. 2009). It is known that, after oral administration, longer-chain 1-alkanols are oxidized to their respective acids in the cells of the small intestines. These are then absorbed and further metabolized in the liver (IFA 2020).

After oral administration of radioactively-labelled 1-hexadecanol (mixture of 1-^3^H-hexadecanol and 1-^14^C-hexadecanol), up to 23% of the radioactivity was found in the plasma in the form of unchanged fatty alcohols. Significant amounts were detected in the form of phospholipids, triglycerides and diacyl glyceryl ether. A small amount was recovered as free fatty acids and wax esters. The study did not provide exact figures for the amounts of radioactivity recovered (Veenstra et al. 2009).

According to in vitro models and experimental studies with hairless mice, the dermal absorption of alcohols decreases with increasing chain length; however, there are no quantitative data. An in vitro skin penetration study with donated human skin samples (n = 3) and a radioactively-labelled 2% tetradecanol emulsion in oil/water was carried out according to OECD test guidelines, but not in compliance with GLP criteria. The emulsion corresponds to a typical body lotion. 1-Tetradecanol was chosen for the study because it is the shortest long-chain alcohol used in personal hygiene products and the level of absorption is therefore expected to be the highest (worst case). Dermal absorption decreases with increasing chain length because of the decreasing water solubility and increasing molar mass. For example, the skin penetration coefficient of 1-hexadecanol is lower than that of 1-tetradecanol by a factor of 2.7 (calculated according to the DERMWIN model Epi Suite v. 3.12; US EPA 2005). The penetration of 1-tetradecanol, applied as a 2% 1-tetradecanol emulsion in a concentration of 2 mg/cm^2^, was determined to be 32% within 24 hours (Veenstra et al. 2009). This is equivalent to a dermal flux of 1.07 mg of 1-tetradecanol per 2000 cm^2^ in 1 hour.

### 3.2 Metabolism

In vivo, primary aliphatic alcohols are generally either oxidized to carboxylic acid via the aldehyde or directly conjugated with glucuronic acid. The aldehyde may be intercepted by glucuronic acid during the oxidation reaction to carboxylic acid and eliminated in the form of an ester. Carboxylic acids may be metabolized further by mitochondrial β-oxidation via acetyl CoA intermediates which involves the stepwise cleavage of C_2_ units. The faster that oxidation to carboxylic acid takes place, the smaller the amount of alcohol that undergoes glucuronidation, unless the doses involved are very high. The carboxylic acids of longer chain aliphatic alcohols may also undergo lipid metabolism and be metabolized to phospholipids or neutral lipids (IFA 2020; Veenstra et al. 2009).

## 4 Effects in Humans

Data for humans are available only for sensitization.

### Allergenic effects

#### Sensitizing effects on the skin

No data are available for the mixture of C_12–18_ fatty alcohols [67762-25-8], but studies were carried out with individual constituents of the mixture. However, the currently available sources only rarely include information about the purity of the fatty alcohols used for testing.

Overall, in view of their widespread distribution, the number of contact allergies the C_12–18_ fatty alcohols are known to have caused is very small (Table 2 and 3; Greim 2000, 2001 a, b, c). Cetyl stearyl alcohol (cetearyl alcohol, mixture of C_16_ and C_18_) is included as a 20% formulation in the standard series “Externa-Inhaltsstoffe” (“topical preparations/ingredients”) of the German Contact Allergy Group (Deutsche Kontaktallergie-Gruppe, DKG). No other fatty alcohols are included in any of the standard series used in Germany.

##### Studies relating to occupational exposure

No studies with occupational exposure were available to the Commission in the past for the evaluation of lauryl alcohol (Greim 2000), myristyl alcohol (Greim 2001 c), cetyl alcohol (Greim 2001 a) and stearyl alcohol (Greim 2001 b).

In a study that focused on glove materials as well as hand disinfectants, soaps and creams, patch tests were performed on a total of 502 hospital workers using a 5% formulation of myristyl alcohol (purity not specified). Data were evaluated for 425 persons working in nursing, 311 of whom had hand eczema in the year of the study. Three of 311 persons (1%) produced positive reactions at the reading on day 3 and several reacted again on day 7; these included 1 person with occupational contact dermatitis. A collective of 114 persons who worked in nursing, but did not have hand eczema, was used for comparison; none of these persons reacted to myristyl alcohol. Particularly noteworthy was the extremely high number of questionable reactions produced by 81 of the 502 (16%) persons in total who were tested in the study. However, there are no data for positive reactions produced by hospital workers who were not involved in nursing (Hamnerius et al. 2018).

In a study with 144 metal workers who were suspected of having occupational dermatitis, 4 persons (2.8%) reacted to 10% myristyl alcohol (purity 95%–100%) with a 1+ reaction on day 3. The authors interpreted the reactions as irritation. In addition, 16 questionably positive reactions and 4 irritant reactions were determined. The authors recommend the use of a formulation containing 5% myristyl alcohol for testing (Geier et al. 2006 a).

In a test with cetearyl alcohol (20%), a questionable reaction was observed in 1 of 214 persons suspected of having an allergy to metal-working fluids (Geier et al. 2004).

In a study that investigated emulsifying agents, a total of 50 of 310 consecutively tested persons produced positive reactions to an emulsifying agent (including myristyl, cetyl and stearyl alcohol); for details, see Table 2. The risk of sensitization to emulsifying agents was higher with statistical significance in workers in the healthcare and nursing sectors than in housewives and persons working in the food sector (p < 0.05) (Corazza et al. 2016). However, the persons who had produced positive reactions to fatty alcohols were not classified according to occupational groups.

##### Studies relating to the use of medicines

The highest percentage of positive reactions (5%–8%) to cetyl, stearyl and cetearyl alcohol were reported by studies that were carried out to investigate medicines. The patients were eczema patients or patients with venous ulcers or stasis eczema on the lower legs. The large number of positive reactions produced in consecutive patch tests by patients with pre-existing eczema may, on the one hand, have been due to a lower irritation threshold that, in some cases, may have led to false-positive irritant reactions. On the other hand, the studies that used consecutive testing did not provide any information about the actual clinical relevance of the reactions that were detected. In addition, these patients had a damaged skin barrier because of their condition; this damage would have promoted the penetration of potential allergens and thus sensitization (Greim 2001 b; Voller et al. 2021).

Tests carried out with 20% formulations of cetearyl alcohol yielded positive reactions in 4.4% to 7.5% of the cases (for example: Valois et al. 2015; 16 of 354 positive reactions (4.5%), Information Network of Departments of Dermatology (IVDK) evaluation with 361 of 4816 (7.5%) and 223 of 5062 (4.4%), respectively) (Erfurt-Berge et al. 2017).

A high incidence of reactions was likewise found in persons being tested for intolerance reactions to a cream containing 5-fluorouracil that is used to treat actinic keratosis. Three of 14 persons produced 2+ reactions to cetearyl alcohol (30%, purity not specified). In 2 of the 3 cases, however, positive reactions were obtained also for other allergens. Only 1 patient reacted exclusively to cetearyl alcohol and the cream (Meijer and de Waard-van der Spek 2007).

In a study, patch tests were performed in patients using a special series for topical medicines. Two of 215 (0.9%) persons reacted to 20% cetyl alcohol and 2 of 215 (0.9%) to 20% stearyl alcohol (Knijp et al. 2019). Positive reactions to cetearyl alcohol (20%, purity not specified) were obtained in 15 of 773 (1.94%) persons (Horton et al. 2021) and in 6 of 407 (1.5%) persons tested with a special series. Another 4.2% produced questionable or irritant reactions (Knijp et al. 2019).

##### Studies relating to the use of cosmetics or with consecutively tested persons

Studies carried out with cosmetics or with consecutively tested persons without pre-existing conditions yielded a much lower percentage of reactions (0.25%–0.8% for cetyl, stearyl and cetearyl alcohol; 3.5% for myristyl alcohol). However, the clinical relevance of the reactions was not evaluated in these studies.

###### Lauryl alcohol (C_12_)

Patch tests were performed with lauryl alcohol (10%) in 21 persons suspected of having allergic contact dermatitis caused by epilation products (wax and tissue). Three of the 21 persons produced strong positive reactions (++/+++) and 5 others 1+ reactions. No information was provided about the severity of the reaction observed in 1 person (see Table 2). All persons who produced positive reactions to lauryl alcohol reacted to at least one other constituent of the epilation products (colophonium derivatives, PEG copolymers, fragrance mixes) (Goossens et al. 2002).

###### Myristyl alcohol (C_14_)

In a study that investigated emulsifying agents (for details see Table 2), 11 of 310 (3.5%) persons reacted to a test formulation of 5% myristyl alcohol (four 1+ and seven 2+ reactions) (Corazza et al. 2016).

In a study that tested 90 consecutive patients with a 10% formulation of myristyl alcohol (purity 99%), 6 persons produced a 1+ reaction and 35 persons a questionable positive reaction at the readings on day 3. The values of the reaction index RI (defined as the quotient of (a – d – i) / (a + d + i); where: a = the number of allergic reactions, d = the number of questionable reactions, i = the number of irritant reactions (Brasch and Henseler 1992)) and the positivity index PI (defined as the percentage of 1+ reactions of the total number of positive reactions (Geier et al. 2003)) (RI = –0.7 and PI = 100%) suggest that the formulation of allergens used for testing was problematical. None of the cases had clinical relevance. Therefore, it may be necessary to interpret the 1+ reactions as false positive or irritant reactions. The 10% formulation was not considered suitable because it caused irritation. As a result, the authors recommend the use of a 5% formulation for testing (Geier et al. 2006 b).

###### Cetyl alcohol (C_16_)

In a study, patch tests were performed in 2825 persons to investigate the effects of 5% cetyl alcohol. Positive reactions were not obtained in any of the tested persons (Horton et al. 2021). In a study that used a much higher test concentration (30%), 2 of 310 (0.6%) consecutively tested persons produced a 1+ reaction (for details see Table 2) (Corazza et al. 2016).

###### Stearyl alcohol (C_18_)

Positive reactions were obtained in 2 of 785 (0.25%) persons tested with 30% stearyl alcohol (Horton et al. 2021). In a test carried out to investigate emulsifying agents (for details see Table 2), 311 persons did not produce a reaction to 30% stearyl alcohol (Corazza et al. 2016).

###### Cetearyl alcohol (C_16/18_ mixture)

Cetearyl alcohol (C_16_ and C_18_) was tested as a 20% formulation within the DKG standard series “Externa-Inhaltsstoffe” (“topical preparations/ingredients”). The percentage of persons tested with the standard series who reacted to cetearyl alcohol (20%) was between 0.13% (1 of 748, Bizjak et al. 2022) and 0.8% (24 of 2967, Britton et al. 2003; 339 of 42 888 (0.79%), Horton et al. 2021; 1045 of 124 517 (0.84%), Uter et al. 2020)). A review of the data collected for a total of 124 517 persons between 2007 and 2018 and evaluated in 3 periods of 4 years did not find a trend in the reaction incidence (Uter et al. 2020).

##### Individual cases

Individual cases of occupational contact dermatitis caused by exposure to fatty alcohols have not been reported to date.

However, there are several reports of patch tests that resulted in positive reactions to stearyl alcohol, cetyl alcohol and cetearyl alcohol (see Table 3). The vast majority of the reported cases involve reactions to topical formulations used to treat skin diseases; the skin barrier of the patients was thus already damaged. Isolated positive results were obtained in tests with low concentrations (5%–10%) of cetyl alcohol and myristyl alcohol. Positive reactions were observed also with higher concentrations (up to 30%) of cetyl alcohol. Tests with 30% stearyl alcohol yielded reactions from 1+ to a maximum of 2+. Most patch tests were carried out with a 20% formulation of a cetearyl alcohol mixture. Overall, these tests yielded reactions from 1+ to a maximum of 2+. Suspected cross-reactions within the group of fatty alcohols (for example stearyl and cetyl alcohol) and cross-reactions between cetyl alcohol and, for example, cetyl palmitate, cetyl lactate and palmitic acid or lactic acid were reported (Voller et al. 2021).

##### Summary

Overall, the C_12–18_ fatty alcohols are compounds with extremely low sensitization potential. The positive reactions in patch tests were obtained mainly from cases with previous damage to the skin.

**Tab. 2:** Studies with patch tests carried out with fatty alcohols in patients with a suspected contact allergy that were published as of 1998

**Concentration test substance^a)^, in petrolatum**	**Tested persons**	**Results:** **reactions**	**Comments**	**References**
**Occupational exposure**				
5% myristyl alcohol	425, of these:		**Test period**: no data, **Collective**: 502 hospital workers were tested with myristyl alcohol. Positive results were reported and evaluated for a total of 425 persons working in nursing professions:	Hamnerius et al. 2018
	311	3 (1%)	311 of them (270 ♀, 41 ♂, average age: 43 years) had developed hand eczema within 12 months, 1/3: occupational contact dermatitis	
	114	0	Comparison group: 114 persons (92 ♀, 22 ♂, average age: 46 years) were tested who worked in nursing professions and did not have hand eczema. Tests were carried out with constituents of the gloves, hand disinfectants, soaps, moisturizing creams. **Reading time and results**: days 3/4 and 7 Questionable reactions to myristyl alcohol were produced by 81 of a total of 502 tested persons. No data for positive reactions produced by persons not working in nursing professions.	
10% myristyl alcohol	144	4 (2.8%)	**Test periods**: 2000–2002 and 2004–2005, **Collective**: IVDK study, 144 metal workers (9 ♀, 135 ♂, 19–62 years, average age: 40 years) who handled metal-working fluids (54 machinists, 49 metal workers (not specified in more detail), 19 lathe operators, 7 toolmakers, 5 grinders and 10 former metal workers who had a different occupation at the time of examination). 29 persons were atopic, 117 had hand dermatitis, 111 were diagnosed with occupational dermatitis. Test substances were standard series allergens and components of metal-working fluids. **Reading time and results**: day 3 (in exceptional cases day 4), 4 × 1+, evaluated as irritant reactions by the authors, 16 × ?, 4 × irritation The authors recommend the use of a more diluted formulation for patch testing because the 10% formulation caused irritation.	Geier et al. 2006 a
20% cetearyl alcohol	214	0	**Test period**: 2002–2003, **Collective**: Of a total of 16 848 patients who underwent patch testing during the specified time period, 251 metal workers were suspected of having an allergy to metal-working fluids. 214 of these workers were tested with cetearyl alcohol. **Reading time and results**: day 3, 0.0% (0.0–1.4), 1 × ?	Geier et al. 2004
5% myristyl alcohol	310	11 (3.5%)	**Test period**: 8 months (no other data), **Collective**: 310 patients (220 ♀, 90 ♂) who were tested for allergies during the test period. **Reading time and results**: days 2, 3 Workers in healthcare and nursing professions had a significantly higher risk of sensitization to emulsifying agents in comparison with that of housewives and persons working in the food industry (p < 0.05). Persons who reacted to fatty alcohols were not classified by occupational groups. 4 × 1+, 7 × 2+	Corazza et al. 2016
30% cetyl alcohol	310	2 (0.5%)	2 × 1+	
30% stearyl alcohol	310	0		
**Medicines**				
20% cetearyl alcohol	354	16 (4.5%)	**Test period**: 42 months, **Collective**: 354 patients (226 ♀, 128 ♂, average age: 75 years) with chronic ulcus cruris (ulcers on the lower leg) were tested with a standard series, special series and wound dressings/plasters. **Reading time**: day 2, 4 or 5	Valois et al. 2015
20% cetearyl alcohol	4816	361 (7.5%)	**Test period**: 1994–2003, **Collective**: IVDK study with 4881 patients (3246 ♀, 1635 ♂, median age: 72 years) with dermatitis of the lower legs, chronic venous insufficiency or chronic leg ulcers; 4816 of the patients were tested with the baseline series (incl. cetearyl alcohol). **Reading time and results**: day 3, 95% CI: 6.8–8.3	Erfurt-Berge et al. 2017
20% cetearyl alcohol	5062	223 (4.4%)	**Test period**: 2003–2014, **Collective**: IVDK study with 5264 patients (3158 ♀, 2106 ♂, 48–88 years, median age: 73 years) with dermatitis of the lower legs, chronic venous insufficiency or chronic leg ulcers; 5062 of the patients were tested with the baseline series (incl. cetearyl alcohol). **Reading time and results**: day 3/4, 95% CI: 3.9–5.0 The patients were tested also with lanolin alcohol (7.8% positive). The 76 patients who reacted to both lanolin alcohol and cetearyl alcohol represent 19% of those who reacted to lanolin alcohol and 35% of those who produced positive reactions to cetearyl alcohol. Control group without this diagnosis: 0.7% (0.6–0.8) n = 55 510	Erfurt-Berge et al. 2017
30% stearyl alcohol	14	3	**Test period**: 2004–2006, **Collective**: 14 patients (7 ♀, 7 ♂, 51–80 years old) suspected of having a contact allergy to a cream containing 5-fluorouracil. **Reading time and results**: days 2, 4, 7, 2+ reaction at each reading, 2/3 produced positive reactions also to 5-fluorouracil, propylene glycol and the cream, 1/3 reacted only to the cream and stearyl alcohol.	Meijer and de Waard-van der Spek 2007
**Cosmetics and consecutively tested persons**				
10% lauryl alcohol	21	9	**Test period**: June 2000–December 2001, **Collective**: of 33 cases of acute dermatitis caused by the use of epilation products (containing ingredients such as lauryl alcohol), 26 female patients (16–42 years old) were tested with the standard series and the epilation products; 21 of these patients were tested with lauryl alcohol. **Reading time and results**: 2 readings, no other data, 2+/2+, –/1+, 3+/2+, –/1+, 1+/–, 1+/1+, –/1+, “positive”/“positive”, 2+/ questionable irritation All female patients who produced positive reactions to lauryl alcohol, reacted also to at least one other ingredient. One of the 21 female patients was tested with a 4% formulation; no reaction was observed.	Goossens et al. 2002
10% myristyl alcohol	90	6	**Test period**: no data, **Collective:** 90 consecutive, not selected patients were tested with a standard series and additionally with myristyl alcohol (possible overlap with the collective of Geier et al. 2006 a). **Reading time and results:** day 3, 6 × 1+, 35 × ? The authors recommend that patch tests with myristyl alcohol should use a 5% formulation because a 10% formulation caused irritation.	Geier et al. 2006 b
5% cetyl alcohol	106	1 (1%)	**Test period**: no data, **Collective**: consecutively tested patients (no other data) suspected of having a contact allergy to cosmetics. **Reading time and results**: no data 1 × 1+ reaction to cetyl alcohol	Guin 2005
30% stearyl alcohol	36	0	1 × reaction to stearyl alcohol	
20% cetearyl alcohol	748	1 (0.13%)	**Test period**: 2019–2021, **Collective**: 748 consecutively tested patients (550 ♀, 198 ♂, median age: 45 years) suspected of having allergic contact dermatitis were tested with various standard allergens. **Reading time and results**: days 3, 6/7 1 person had a decrescendo reaction.	Bizjak et al. 2022
20% cetearyl alcohol	2967	24 (0.8%)	**Test period**: within 1 year (2000), **Collective**: 2967 patients (2763 ♀, 204 ♂) from 6 centres who were suspected of having allergic contact dermatitis were tested with various standard allergens. **Reading time and results**: days 2, 4, % positive reactions from each centre: 0.9 (n = 585); 0.5 (n = 383); 1.8 (n = 674); 0.6 (n = 669); 2.0 (n = 260); 0.0 (n = 396); mean of 0.8%, 95% CI: 0.2–1.4, additional irritant reactions at one of the centres: 0.5%	Britton et al. 2003
20% cetearyl alcohol	407	6 (1.5%)	**Test period**: January 2016–December 2017, **Collective**: of a total collective of 594 patients who were tested using a standard series, 407 were additionally tested with a wool alcohol series and 215 with a topical medicine series. **Reading time and results**: days 2 and 3/4, in some cases day 6/7 cetearyl alcohol: 95% CI: 0.3–2.6 additionally 17 × ? and irritant reactions	Knijp et al. 2019
20% cetyl alcohol	215	2 (0.9%)	95% CI: 0–2.2, additionally 3 × ?	
20% stearyl alcohol	215	2 (0.9%)	95% CI: 0–2.2, additionally 6 × ?	
20% cetearyl alcohol	214	1 (0.5%)	95% CI: 0–1.4, additionally 7 × ?, possible overlap with patients from wool alcohol series	
20% cetearyl alcohol	42 888	339 (0.79%)	**Test period**: 2009–2018, **Collective**: evaluation of ESSCA data; patients who were tested with cetearyl alcohol within a standard series. **Reading time and results**: no data, (95% CI: 0.7–0.87), 1+: 0.68%, 2+/3+: 0.27%, ?: 1.16%, irritation: 0.71%, positive reactions categorized by country: Germany: n = 191 660 (0.95%, 95% CI: 0.82–1.1), Austria: n = 2367 (1.06%, 95% CI: 0.68–1.56), Switzerland: n = 12 154 (0.93%, 95% CI: 0.77–1.12), UK: n = 8707 (0.14%, 95% CI: 0.07–0.24)	Horton et al. 2021
	773	15 (1.94%)	In Spain and the Netherlands, cetearyl alcohol is included in a special series (95% CI: 1.09–3.18): +: 1.68%, 2+/3+: 0.2%, ?: 3.31%, irritation: 0%, positive reactions categorized by country: Spain: n = 86 (1.16%, 95% CI: 0.03–6.31), Netherlands: n = 687 (2.04%, 95% CI: 1.12–3.4)	
30% stearyl alcohol	785	2 (0.25%)	In Spain, tested in a special series (95% CI: 0.03–0.92): no data for the severity of the reactions	
5% cetyl alcohol	2825	0	In Spain and the Netherlands, tested in a special series (95% CI: 0.03–0.92): of these: Spain: n = 785 (0%, 95% CI: 0–0.38), Netherlands: n = 2040 (0%, 95% CI: 0–0.15), no data for the severity of the reactions	
20% cetearyl alcohol	124 517, of these:	1045 (0.84%)	**Collective**: IVDK evaluation of patch test results grouped in 3 periods of 4 years (see below). Of a total collective of 141 762 patients, 125 436 (79 395 ♀, 46 041 ♂, in total 72% ≥ 40 years old) were tested using a standard series. 21 096 persons were diagnosed with occupational dermatitis. 124 517 persons in total were tested with cetearyl alcohol. **Reading time and results**: day 3 (day 4)	Uter et al. 2020
	43 056	362 (0.84%)	**Test period**: 2007–2010, 95% CI: 0.75–0.93	
	44 385	457 (1.03%)	**Test period**: 2011–2014, 95% CI: 0.93–1.12	
	37 076	226 (0.61%)	**Test period**: 2015–2018, 95% CI: 0.53–0.69	
cetearyl alcohol (probably 20%)	1537	0.5%	**Test period**: 1997–1998, **Collective**: study carried out by the Kooperative Gesundheitsforschung in der Region Augsburg (KORA), (possible overlap of the collective). **Reading time**: no data	Uter et al. 2002
cetearyl alcohol (probably 20%)	555	3.8%	**Test period**: 1997–2000, **Collective**: IVDK study, **Reading time**: no data	

CI: confidence interval; ESSCA: European Surveillance System on Contact Allergies; IVDK: Information Network of Departments of Dermatology; KORA: Kooperative Gesundheitsforschung in der Region Augsburg (Cooperative Health Research in the Region of Augsburg); ?: questionable reaction

^a)^ purity only specified if known

**Tab. 3:** Positive single findings in patch tests carried out with fatty alcohols in patients with a suspected contact allergy caused by cosmetics or medicines that were published as of 1998 (after completion of data collection for Greim 2000 (1-Dodecanol), Greim 2001 a (1-Hexadecanol), Greim 2001 c (1-Tetradecanol), Greim 2001 b (1-Octadecanol))

**Test substance^a)^,** **concentration, in petrolatum^b)^**	**Results:** **reactions**	**Comments**	**References**
**Suspected contact allergy caused by medicines (damaged skin barrier)**			
5% cetyl alcohol	1+ (days 2, 3)	60-year-old male patient with pruritic eczematous eruption after treatment for tinea pedis (athlete’s foot) using a topical formulation. After positive results were obtained in patch tests with this topical formulation and with other anti-fungal creams, ingredients of the creams were tested. Positive reactions to, among others, lanoconazole (active ingredient) and diethyl sebacate (ingredient of the cream)	Soga et al. 2004
30% stearyl alcohol	1+ (day 3) ? (day 7)	69-year-old male patient with psoriasis developed dermatitis on his lower legs and arms after using moisturizing creams and topical corticosteroids; additional positive reactions to, among others, diazolidinyl urea, imidazolindinyl urea, various fragrances, dichlorobenzyl alcohol and the emulsifying agents stearyl alcohol, steareth-7 and steareth-10	Thormann et al. 2009
stearyl alcohol (no other data)		3 female patients who were prescribed a cream containing 5-fluorouracil for the treatment of skin disorders: 3/3 reacted to the cream, but did not produce positive reactions to 5-fluorouracil and other ingredients of the cream included in the test	Yesudian and King 2001
	2+ (days 2, 4)	72-year-old female patient with Bowen’s disease (precursor for skin cancer): sudden severe inflammation after use for 5 months	
	2+ (days 2, 4)	66-year-old female patient following treatment of actinic keratosis. After first treatment with the cream, immediate improvement of the keratosis symptoms. Eczematous reaction after renewed application when symptoms reocurred 4 months later	
	2+ (days 2, 4)	57-year-old female patient with Bowen’s disease: inflammatory reaction after treatment for 3 weeks	
30% cetyl alcohol	1+ (days 3, 4)	60-year-old female patient with pruritic erythematous macules on the neck and inside of the elbow, areas where she had applied a topical formulation. Also positive results in patch tests with the cream and the ingredient crotamiton	Oiso et al. 2003
20% cetearyl alcohol	1+	61-year-old female patient with dermatitis on her ankle after treatment with a first aid cream; several of the cosmetic products she used contained cetyl stearyl alcohol. 2+ reaction to doxepin cream, 1+ reaction to the cream she used that contained cetyl alcohol, no reaction to diphenhydramine (1 % in petrolatum), no data for the time point when the reading was taken	Aakhus and Warshaw 2011
20% cetearyl alcohol	1+ (day 3)	53-year-old housewife with dermatitis on her face after self-prescribed treatment with a formulation containing cetrimide and cetearyl. Also positive reactions to the formulation (2+) and cetrimide (0.1% aqueous) (1+)	Leow and Tan 2000
20% cetearyl alcohol	1+ (day 2) – (days 4, 7)	36-year-old female patient with dermatitis on her face and eyelids, dermatitis developed also after applying a corticosteroid cream to a leg ulcer. Several cosmetic products and the corticosteroid cream of the patient contained steareth-10 and small amounts of stearyl alcohol (50 ppm in wet wipes). Positive reactions (1+) also to the corticosteroid cream (day 2) and to steareth-10 (day 4, 5% aqueous)	Aerts et al. 2017
10% stearyl alcohol	?		
20% cetearyl alcohol	1+ (day 3)	75-year-old female patient with foot dermatitis that had persisted for more than 1 year, treatment of a flare-up with thrombocid cream, 24 hours later, severe erythema and blistering. Positive reactions to mercury (2+), potassium dichromate (2+), caine mix (2+), clioquinol (1+) in addition to the cream (2+) and Emulgade F (2+, contains cetearyl alcohol), a control group of 18 persons produced negative results	Armengot-Carbo et al. 2016
	2+ (day 3)	82-year-old female patient with dermatitis after treatment of chronic oedema of the lower leg and venous insufficiency with a thrombocid cream; positive reactions to the cream (2+), a fragrance mix (2+) and Emulgade F (2+, contains cetearyl alcohol), a control group of 18 persons produced negative results	
20% cetearyl alcohol	“positive” (day 3)	49-year-old male patient with eczema on the torso and limbs about 10 days after treatment of abrasions with a cream containing dexpanthenol. Reactions also to dexpanthenol and the cream	Miroux-Catarino et al. 2019
30% stearyl alcohol (in 5% aqueous propylene glycol solution)	+ (day 2) 2+ (day 4)	48-year-old female patient with an itchy, erythematous spot on an eyelid after applying an eye cream for the treatment of seborrheic dermatitis, worsening of the lesions. The patient produced a positive reaction to neomycin sulfate, an ingredient of the cream. Additionally, creams used for the treatment of dermatitis were tested; the patient reacted to 2 of these creams. The ingredients of both creams were subsequently tested (both contained, among other ingredients, stearyl alcohol and cetyl alcohol). The patient reported having developed a skin rash in the past after applying cosmetics	Kang et al. 2004
30% cetyl alcohol (vehicle see above)	– (days 2, 4) 1+ (days 2, 4)		
		7 persons (4 ♀; 3 ♂ between 36 and 72 years) with dermatitis caused by topical medicines that contained stearyl alcohol and in some cases cetyl alcohol (between 2013 and 2019), 3 control persons tested with stearyl alcohol (30%) produced negative results (days 2, 3 and 7)	Nishioka et al. 2022
30% stearyl alcohol	1+ (days 2, 3, 7)	50-year-old male patient	
30% cetyl alcohol	– (days 2, 3, 7)		
30% stearyl alcohol	1+ (day 4) ? (day 7)	62-year-old male patient, no reaction to the suspected formulation	
30% stearyl alcohol	1+ (days 2, 3)	44-year-old female patient, day 7 no reading taken	
30% stearyl alcohol	1+ (days 2, 3)	36-year-old female patient, day 7 no reading taken	
30% stearyl alcohol	1+ (days 2, 3) ? (day 7)	56-year-old female patient	
30% cetyl alcohol	1+ (days 2, 3) ? (day 7)	products contained also cetyl alcohol	
30% stearyl alcohol	1+ (days 2, 3, 7)	54-year-old male patient	
30% cetyl alcohol	? (day 2) 1+ (day 3) – (day 7)	products contained also cetyl alcohol	
30% stearyl alcohol	? (day 2) 1+ (day 3) – (day 7)	48-year-old female patient, products contained also cetyl alcohol (but not tested)	
5% cetyl alcohol	– (days 2, 4)	57-year-old female patient with psoriasis and eczema on the back of her hands 2 weeks after beginning treatment with a cream containing calcipotriol; a similar rash had developed following application of a different cream 3 years previously	Navarro-Triviño and Ruiz-Villaverde 2022
30% stearyl alcohol	– (days 2, 4)		
20% cetearyl alcohol	2+ (day 2) 3+ (days 4, 7)	reaction also to the cream	
30% stearyl alcohol	1+ (days 2, 4)	61-year-old male patient with extensive pruritic, scaly dermatitis that had persisted for 4 years and had begun on one foot after the use of topical medicines. A biopsy found spongiotic dermatitis with eosinophils. Patch tests were carried out with the standard series and special series (including emulsifying agents, fragrances) and the topical medicines used. Positive reactions to, among others, formaldehyde, methylisothiazolinone and questionable reactions to cetearyl glycoside. Subsequent testing of the inactive ingredients of the cream	Ruggiero et al. 2021
30% cetyl alcohol	– (days 2, 4)		
**Suspected contact allergy caused by cosmetics, etc. (intact skin barrier)**			
5% cetyl alcohol	2+ (days 2, 4)	24-year-old female patient with dermatitis caused by a face cream, positive reactions to the face cream and benzophenone-3, photoallergic reactions to benzophenone-3 and benzophenone-10	Kiec-Swierczynska et al. 2005
5% cetyl alcohol	? (day 4 or 6)	66-year-old female patient with dermatitis in the armpit, on the arms, abdomen, chest and upper thighs that had persisted for 5 months. A skin biopsy of the chest found spongiotic dermatitis with eosinophils. Self-prescribed treatment included a hydrocortisone cream. Tests included the allergens of a standard series and, among others, a medicine, textile and cosmetic series in addition to personal products of the patient. Testing found positive reactions to, among others, dipropylene glycol, nickel, formaldehyde, dye mix and several personal products. Dipropylene glycol in cosmetics was determined as the cause	Peterson et al. 2022
20% cetearyl alcohol	1+ (day 4 or 6)		
30% stearyl alcohol	1+ (day 4 or 6)		
stearyl alcohol (no other data)	“strong positive” days 2, 4	65-year-old male patient who had formerly worked as a farmer with oedematous and vesicular dermatitis on the back of the hand, wrist and lower arm after wearing rubber gloves coated with a moisturizing formulation to apply ammonia water while gardening. The symptoms worsened after treatment of the dermatitis with a topical corticosteroid. Improvement after administration of oral steroids, systemic antihistamines, topical corticosteroids and ibuprofen. Subsequent treatment with a moisturizing cream caused a flare-up of the dermatitis. Previously diagnosed allergies to cetrimide (quaternary ammonium compound), isopropyl alcohol, iodine-containing compounds (allergies had developed several years before during the treatment of an elbow injury). The patient had worn rubber gloves for more than 20 years without complications while working as a farmer. First set of tests included the gloves (strong positive reaction) and a standard series (positive reaction only to cetearyl alcohol). Both the corticosteroid cream and the moisturizing cream contained cetearyl alcohol. An extraction from the rubber gloves showed that the gloves contained different fatty alcohols (C_12_, C_18_ and C_20_) and a quaternary ammonium compound. Another set of patch tests was carried out with various fatty alcohols and ammonium compounds (also strong positive reactions). According to the authors, the sensitization to fatty alcohols appears to have been caused by the past use of cetyl-containing creams	Vanden Broecke et al. 2014
cetyl alcohol (no other data)	“strong positive” days 2, 4		
behenyl alcohol (no other data)	“strong positive”	behenyl alcohol contains lauryl (C_12_), myristyl (C_14_), cetyl or palmityl alcohol (C_16_) in addition to stearyl (C_18_) and oleyl alcohol (C_18_ unsaturated)	
5% myristyl alcohol	1+ (days 2, 3)	44-year-old female patient with dermatitis caused by a deodorant, positive reactions to ceteareth-2 and ceteareth-3 (no reaction to ceteareth-20, 25 and 30)	Corazza et al. 2013
30% cetyl alcohol	1+ (day 2) 2+ (day 3)		
30% cetearyl alcohol	1+ (days 2, 3)		
30% stearyl alcohol	1+ (days 2, 3)		
20% cetearyl alcohol	2+	62-year-old female patient with pre-existing hand dermatitis that worsened considerably over a period of 2 years. Several cosmetics the patient used contained cetyl alcohol. 3+ reaction to one of the moisturizing creams the patient used that contained cetyl alcohol no data for the time point when the reading was taken	Aakhus and Warshaw 2011

1+, 2+, 3+: severity of the reaction; ?: questionable reaction

^a)^ purity only specified if known

^b)^ unless specified otherwise

#### Sensitizing effects on the airways

There are no studies available.

## 5 Animal Experiments and in vitro Studies

### 5.1 Acute toxicity

#### 5.1.1 Inhalation

There are no experimental studies available.

As the fatty alcohols with chain lengths from C_12_ to C_22_ are of low volatility, only low concentrations can be achieved in the vapour phase (for example, a 1-dodecanol concentration of 1 mg/m^3^ or a 1-octadeconal concentration of 0.01 mg/m^3^). Therefore, toxicity is not expected to occur after exposure to an atmosphere saturated with vapour. The LC_50_ values of all fatty alcohols tested (no other details) exceeded the saturation concentration (Veenstra et al. 2009).

As the RD_50_ values for 1-heptanol and 1-octanol are in a range of 50 to 100 ml/m^3^ in mice (no other details), longer-chain fatty alcohols, which have a much lower vapour pressure and thus occur in the air as a vapour in much lower concentrations, are assumed to pose a low risk for sensory irritation (Veenstra et al. 2009).

#### 5.1.2 Oral administration

An experimental study carried out in 1981 established an oral LD_50_ > 5000 mg/kg body weight for fatty alcohols of type A in Wistar rats (male and female animals). Fatty alcohols of type B are likewise expected to have low toxicity (OECD 2006).

1-Hexanol has an oral LD_50_ of 3000 to 4000 mg/kg body weight; the acute oral toxicity of alcohols with chain lengths of C_8_ and higher lies above this range (no other data; Veenstra et al. 2009).

#### 5.1.3 Dermal application

There are no experimental studies available for this end point.

On the basis of the findings of studies that investigated alcohols with similar chain lengths, the fatty alcohols are expected to have low acute dermal toxicity and an LD_50_ of > 2000 mg/kg body weight without any signs of systemic toxicity (OECD 2006; Veenstra et al. 2009).

### 5.2 Subacute, subchronic and chronic toxicity

There are no studies available that investigated C_12–18_ fatty alcohols.

#### 5.2.1 Inhalation

No adverse effects were observed after 9-day exposure of groups of 8 male and 8 female F344 rats for 6 hours a day to a saturated atmosphere of C_9–11_ alcohols (CAS No. 66455-17-2, > 80% linear C_9_, C_10_, C_11_ alcohols) in a concentration of about 158 mg/m^3^. Clinical observations, body weights, clinico-chemical and haematological parameters, gross-pathological necropsy, organ weights and a histopathological investigation of the respiratory tract in addition to all lesions detected by gross pathology were included in the examination and compared with the findings in the control group (no other details; OECD 2006; Veenstra et al. 2009).

#### 5.2.2 Oral administration

Studies with repeated oral administration of various long-chain fatty alcohols in rats (no other details) derived a NOAEL (no observed adverse effect level) of more than 100 mg/kg body weight and day after administration for 90 days and a NOAEL of more than 300 mg/kg body weight and day after administration for at least 28 days. 1-Hexanol, 1-dodecanol, C_10–16_ alcohols (type B), C_14–16_ alcohols (type A), 1-hexadecanol, 1-octadecanol and 1-docosanol (C_22_) were administered. The substances (≥ C_8_) did not induce irritation at the site of first contact and no neurotoxic effects. At high doses (1000 mg/kg body weight and day), several of the alcohols induced changes in clinico-chemical parameters for the liver without any other histopathological findings (OECD 2006).

**Tab. 4:** Toxicity induced by alcohols after repeated oral administration

**Species,** **strain,** **number per group**	**Alcohol** **exposure**	**Results**	**References**
**rat**, no other data	C_10–16_, 28 days, 7 days/week, 0, 100, 300, 1000 mg/kg body weight and day, no other data	**300 mg/kg body weight: ♀: NOAEL**; **300 mg/kg body weight and above**: ♂: relative kidney weights increased without histopathological correlate, **NOAEL**; **1000 mg/kg body weight**: ♂: body weight gains decreased by 10%, ♀: alanine aminotransferase increased by 50%, alkaline phosphatase increased by 40%, cholesterol increased by 30%, no histopathological findings	OECD 2006; Veenstra et al. 2009
**rat**, no other data	C_13_, 2 weeks, 184 mg/kg body weight and day, no other data	only liver and testes examined: no effects on weights, no histopathological changes, peroxisome proliferation or hyperlipidaemia	Veenstra et al. 2009
**rat**, no other data	C_14–16_, 13 weeks, 0, 0.2%, 1%, 5% in the feed (about 0, 180, 900, 4500 mg/kg body weight and day, conversion factor 0.09 according to EFSA 2012), no other data	**about 180 mg/kg body weight: NOAEL**; **about 900 mg/kg body weight and above**: feed consumption decreased because of reduced palatability, body weight gains decreased by 15%, changes in alanine aminotransferase and alkaline phosphatase (no other details), changes in organ weights (no other details), liver weights ↑ (no other details), no histopathological changes, insufficient feed consumption; **about 4500 mg/kg body weight**: body weight gains decreased by 30%, insufficient feed consumption	Veenstra et al. 2009
**rat**, no other data	1-hexadecanol C_16_, 28 days, 7 days/week, gavage, 0, 100, 500, 1000 mg/kg body weight and day	**1000 mg/kg body weight: NOAEL**	Veenstra et al. 2009
**rat**, no other data	1-hexadecanol C_16_, 13 weeks with the feed, 7 days/week, 0, 1%, 2.5%, 5% in the feed (0, about 150, 375, 750 mg/kg body weight and day), highest dose increased to 1500 mg/kg body weight and day during the last 3 weeks	**about 150 mg/kg body weight: NOAEL**; **about 375 mg/kg body weight**: “occasional” reduced feed consumption and body weight gains ↓; **about 750 mg/kg body weight**: feed consumption decreased by 10%–24%, body weight gains decreased by 10%–18%, ♂: relative liver weights increased by 124% without histopathological correlate	OECD 2006; Veenstra et al. 2009
**rat**, no other data	C_16–18_ and C_18_ unsaturated, 28 days, 7 days/week, 850 mg/kg body weight and day	**850 mg/kg body weight: NOAEL**	Veenstra et al. 2009
**rat**, no other data	C_18_, 4 weeks, 5 days/week, gavage, 0, 100, 500, 1000 mg/kg body weight and day	**1000 mg/kg body weight: NOAEL**	OECD 2006; Veenstra et al. 2009
**rat**, Wistar, no other data	C_18_, ♂ about 37 days, ♀ about 63 days, 0, 100, 500, 2000 mg/kg body weight and day, combined repeated dose study/screening test for developmental toxicity	in exposed ♂ animals without dose dependency (parameters not examined in ♀): in plasma: glucose decreased by about 15%, triglycerides decreased by about 37%, cholesterol decreased by about 25%, no histopathological findings; **100 mg/kg body weight: NOEL**; **2000 mg/kg body weight: NOAEL**	Veenstra et al. 2009
**dog**, no other data, 2 ♂ and 2 ♀	C_16_, 13 weeks with the feed, 0, 0,5%, 1,0%, 3% in the feed (0, about 167, 333, 1000 mg/kg body weight and day)	increase in aspartate aminotransferase not dose-dependent, no histopathological findings; **about 1000 mg/kg body weight: NOAEL**	OECD 2006; Veenstra et al. 2009

NOAEL: no observed adverse effect level; NOEL: no observed effect level

#### 5.2.3 Dermal application

There are no studies available.

### 5.3 Local effects on skin and mucous membranes

There are no studies available that investigated the C_12–18_ fatty alcohols.

#### 5.3.1 Skin

Both type A and type B fatty alcohols are expected to cause very mild irritation of the skin (OECD 2006).

On the basis of comparative studies, alcohols with chain lengths of C_12–16_ were classified as mildly irritating to the skin and alcohols with chain lengths of C_18_ and above as not irritating to the skin if applied undiluted to the skin for 4 to 24 hours (Veenstra et al. 2009).

#### 5.3.2 Eyes

Type A and type B fatty alcohols are not expected to induce eye irritation (OECD 2006). The self-assessment by REACH registrants indicates a possible irritant effect on the skin and eyes.; however, no corresponding harmonized classifications have been made (ECHA 2023 a).

Fatty alcohols with chain lengths of C_12_ and higher are not expected to induce eye irritation (Veenstra et al. 2009).

### 5.4 Allergenic effects

#### 5.4.1 Sensitizing effects on the skin

There are no studies available of the C_12–18_ fatty alcohols [67762-25-8]. In the earlier documentations for the individual alcohols, no positive findings from animal experiments were described. Recent studies with the individual substances are not available. The REACH registration dossiers include several animal studies that investigated similar fatty alcohols:

In an LLNA (local lymph node assay; equivalent to the later OECD Test Guideline 429) with CBA/Ca mice, positive results were obtained with a mixture of **C_14–15_ alcohols** [75782-87-5]. Stimulation indices of 0.7, 4.0, 9.9 and 16.0, respectively, were determined for the concentrations 1%, 10%, 25% and 50% in acetone/olive oil (4:1 v/v). Erythema was observed after exposure to the two highest concentrations, which indicates that irritation may also cause increased proliferation. No information was provided about how the concentrations were selected (ECHA 2015).

A maximization test was carried out in 10 Hartley guinea pigs with a mixture of **1-eicosanol (C_20_)** [629-96-9] and **1-docosanol (C_22_)** [661-19-8] in compliance with OECD Test Guideline 406. The intradermal induction was carried out with a 5% formulation of the test substance, epicutaneous induction with a 75% formulation in corn oil. None of the animals reacted when challenged with a 10% formulation of the test substance in corn oil. The test results are thus negative (ECHA 2023 c).

A Buehler test with **1-decanol (C_10_)** in 20 Hartley guinea pigs yielded negative results. The epicutaneous induction was performed with the undiluted test substance in a Hill Top chamber; the remaining test substance was rinsed off after 6 hours of treatment. None of the animals reacted to the challenge with a 25% formulation of the test substance in mineral oil. Mild erythema was observed in 5 and 2 of the 20 animals after 24 and 48 hours, respectively; however, this effect was not evaluated as a positive reaction (ECHA 2023 d). The results of this study do not indicate that any of the individual fatty alcohols have sensitizing potential. This is consistent with the negative results obtained with individual substances that were included in earlier documentation for 1-octadecanol and 1-hexadecanol (Greim 2001 a, b).

A study that investigated a mixture of branched fatty alcohols (**C_16–19_ branched alcohols **[93762-74-4]), by contrast, reported positive results in the LLNA. The test (equivalent to the later OECD Test Guideline 429) was carried out in CBA/Ca mice. Stimulation indices of 0.8, 4.2, 8.2 and 16.2, respectively, were determined for the concentrations 1%, 10%, 25% and 50% in acetone/olive oil (4:1 v/v) (ECHA 2023 b).

### 5.5 Reproductive and developmental toxicity

#### 5.5.1 Fertility

There are no generation studies available.

**1-Dodecanol** and **1-octadecanol** were investigated in combined studies of toxicity and reproductive toxicity that were carried out according to valid test guidelines. Neither substance induced effects in male and female rats after administration with the feed up to a dose of 2000 mg/kg body weight and day. **1-Docosanol** (C_22_) did not induce effects after oral administration in male and female rats at doses up to 1000 mg/kg body weight and day (Veenstra et al. 2009).

No effects on the reproductive organs were observed after oral administration of **1-hexadecanol**, **1-octadecanol**, **1-docosanol** or **C_24–34_fatty alcohols** in rats in doses of up to 1000 mg/kg body weight and day for a period of 1 year (Veenstra et al. 2009).

No effects on the reproductive organs were observed after oral administration of **1-hexadecanol** (C_16_, cetyl alcohol, palmityl alcohol) or **1-octadecanol** (C_18_, stearyl alcohol) in rats or dogs in doses of up to 1000 mg/kg body weight and day for a period of up to 1 year (OECD 2006).

#### 5.5.2 Developmental toxicity

There are no studies with the C_12–18_ fatty alcohols that investigated this end point.

In a combined study of toxicity and reproductive toxicity with repeated oral administration of **1-octadecanol**, the glucose (about 15%), triglyceride (about 37%) and cholesterol (about 25%) levels in the plasma of male animals were reduced, but without dose dependency. These parameters were not examined in the female animals. There were no substance-induced histopathological changes and the NOAEL was the highest dose tested of 2000 mg/kg body weight and day (Veenstra et al. 2009).

On the basis of the repeated dose studies and in view of the structure of the substances, type A and type B fatty alcohols without manifest maternal toxicity are not expected to cause effects in the offspring (OECD 2006).

### 5.6 Genotoxicity

#### 5.6.1 In vitro

Type B fatty alcohols yielded negative results in a bacterial mutagenicity test in Salmonella typhimurium TA98, TA100, TA1535, TA1537 and TA1538 up to a concentration of 2500 µg/plate in the presence and in the absence of a metabolic activation system. Cytotoxicity was observed at the second highest concentration of 500 µg/plate and above. The positive control verified that the test system was functional. On this basis, type A fatty alcohols are likewise not expected to induce mutagenic effects (OECD 2006).

Negative results in mutagenicity tests with Salmonella typhimurium were likewise obtained with other alcohols that are listed in Section 5.2.2 (Veenstra et al. 2009).

C_10–16_ fatty alcohols yielded negative results in chromosomal aberration tests in RL1 cells and in CHO cells (Veenstra et al. 2009).

#### 5.6.2 In vivo

There are no studies available that investigated C_12–18_ fatty alcohols.

**1-Dodecanol**, **1-octadecanol**, **1-docosanol** and **C_24–34_fatty alcohols** yielded negative results in micronucleus tests in the bone marrow of mice (Veenstra et al. 2009).

### 5.7 Carcinogenicity

There are no studies available that investigated the C_12–18_ fatty alcohols.

There is no evidence of a carcinogenic effect of (longer-chain) fatty alcohols, nor is there any structural alert.

## 6 Manifesto (MAK value/classification)

A critical effect may be local effects in the respiratory tract. Because of their low vapour pressure, it is unlikely that fatty alcohols pose a hazard in vapour form; however, exposure to a vapour/aerosol at the workplace is possible should aerosolization occur.

**MAK value. **Inhalation studies that investigated the C_12-18_ fatty alcohols are not available.

The Commission has evaluated a number of longer-chain alcohols. MAK values of 10 ml/m^3^ were established for 1-octanol (C_8_; Greim 2003) and 1-decanol (C_10_; Hartwig and MAK Commission 2022); no MAK values were derived for 1-tetradecanol (Greim 2001 c) or 1-hexadecanol (Greim 2001 a).

After repeated oral administration of long-chain fatty alcohols in rats, a NOAEL of at least 150 mg/kg body weight and day was determined for 1-hexadecanol after administration for 90 days, and of 300 mg/kg body weight and day for C_10–16_ after administration for at least 28 days. 1-Hexanol, 2-ethylhexanol, 1-dodecanol, C_10–16_ alcohols (type B), C_14–16_ alcohols (type A), 1-hexadecanol, octadecanol and 1-docosanol (C_22_) were administered (OECD 2006).

The following toxicokinetic data are taken into consideration for the extrapolation of the NOAEL of 150 mg/kg body weight and day derived for 1-hexadecanol to a concentration in workplace air: the daily exposure of the animals in comparison with the 5 days per week exposure at the workplace (7:5), the corresponding species-specific correction value for the rat (1:4), the assumed oral absorption (100%), the body weight (70 kg) and the respiratory volume (10 m^3^) of the person, and the assumed 100% absorption by inhalation. A possible intensification of the effect after chronic exposure (1:2) and the extrapolation of the data from an animal study to humans (1:2) are taken into account. The concentration in air calculated from this is about 92 mg/m^3^. At a respiratory volume of 10 m^3^ and 100% absorption by inhalation, this results in a systemically tolerable amount of 920 mg.

Exposure to fatty alcohols in these amounts does not pose a risk because the corresponding uptake of fatty acids from fat in foods lies in the gram/kg body weight range (DGE 2015). However, it remains unclear whether emulsifying fatty alcohols taken up via inhalation cause effects in the cell membranes in the lungs. As there are no studies that investigated fatty alcohols in the respiratory tract and fatty alcohols are metabolized to fatty acids in vivo, the C_12–18_ fatty alcohols have been classified in Section II b in analogy to the fatty acids previously evaluated by the Commission and their classification in Section II b. Peak limitation does not apply.

**Prenatal toxicity. **There are no studies available that investigated this end point. As a MAK value has not been derived, the mixture has not been classified in a pregnancy risk group.

**Carcinogenicity. **There are no studies and no structural alert for a carcinogenic effect. For this reason, the C_12–18_ fatty alcohols have not been classified in a category for carcinogens.

**Germ cell mutagenicity. **There is no evidence of genotoxicity and no structural alert for this type of effect. For this reason, the C_12–18_ fatty alcohols have not been classified in a category for germ cell mutagens.

**Absorption through the skin. **The acute toxicity after dermal exposure is low. There are no human or animal studies that investigated quantitative dermal absorption or non-lethal toxic effects after dermal exposure. An in vitro study with application of 2 mg of a 2% 1-tetradecanol emulsion per cm^2^ of human skin reported the dermal penetration of 1.07 mg of 1-tetradecanol under standard conditions (2000 cm^2^, 1 hour). Taking into consideration that the systemically tolerable amount of 920 mg (see section “MAK valueˮ) that was determined for 1-hexadecanol is applicable to the entire substance group, the amount that penetrates through the skin is negligible. As a result, the C_12–18_ fatty alcohols have not been designated with an “H” (for substances which can be absorbed through the skin in toxicologically relevant amounts).

**Sensitization. **No studies have been carried out with the mixture of C_12–18_ fatty alcohols. However, the number of allergic reactions to the individual constituents of the mixture is very small in view of the widespread distribution of the fatty alcohols. Most cases involved patients with a damaged skin barrier. Overall, most animal studies that investigated the individual constituents and structurally similar fatty alcohols reported negative results. As a result, the C_12–18_ fatty alcohols have not been designated with “Sh” (for substances which cause sensitization of the skin). There are no data for sensitizing effects on the airways. Therefore, the mixture has not been designated with “Sa” (for substances which cause sensitization of the airways).
